# Research utilisation in clinical practice: the experience of nurses and midwives working in public hospitals

**DOI:** 10.1186/s12978-021-01095-x

**Published:** 2021-03-15

**Authors:** Asrat Hailu Dagne, H. /Mariam Demewozu Tebeje

**Affiliations:** 1 Department of Midwifery, Debre Tabor University, Debre Tabor, Amhara Region Ethiopia; 2 School of midwifery, University of Gondar, Gondar, Amhara Region Ethiopia

**Keywords:** Research utilisation, Barriers, Supporting factors of research utilisation

## Abstract

**Background:**

Nurses and midwives play a vital role to utilise research in clinical decision-making practice. However, limited support for research utilisation and barriers of research utilisation hamper to utilise up-to-date research findings in clinical practice. Therefore, this study aimed to explore nurses’ and midwives’ experience of research utilisation in public hospitals.

**Methods:**

A qualitative descriptive approach was conducted to explore nurses’ and midwives’ experience of research utilisation in clinical practice within South Gondar Zone public hospitals from January 3 to June 28, 2020. A total of 20 interviewees, 40 participants of FGDs, and 8 observations were considered in the study. Data from the interview, FGD, and observation were imported into NVivo 12 plus to manage and analyze the data using the Computer-Assisted Data Analysis Software Program (CAQDAS). The data were analyzed through thematic content analysis.

**Results:**

Nurses’ and midwives’ experience of using research findings in clinical decision-making emerged as “the non-intentional research utilisation” the main theme. Data analysis produced as “the belief towards research utilisation”, “the limited support for nurses and midwives”, and, “the perceived barriers of research utilisation” as the three themes. Participants believed that the non-use of the primary research was recommended due to fear of accountability for client harm. The limited support for nurses’ and midwives’ experience of research utilisation decrease nurses’ and midwives’ confidence to utilise research in clinical practice. Knowledge, attitude, time mismanagement, and the lack of motivation were perceived barriers to research utilisation. The lack of training and access to systematic review and meta-analysis research findings limited the research utilisation in clinical practice.

**Conclusions:**

The experience of research utilisation indicated that there was limited support for nurses and midwives to utilise research. Nurses and midwives did not utilise research in their clinical practice intentionally. This study identified that knowledge, negative attitude towards research utilisation, lack of training; time mismanagement, and lack of motivation were the perceived barriers to research utilisation. Therefore, the promotion of adopting the research utilisation and training on the identified barriers are mandatory.

**Plain English summary:**

Nurses and midwives play a vital role to utilise research in clinical decision-making practice. However, the limited support for research utilisation and barriers of research utilisation hamper the utilisation of up-to-date research in clinical practice. Therefore, this study aimed to explore nurses’ and midwives’ experience of using the knowledge obtained from research findings in clinical and healthcare decision-making practice within public hospitals.

The experience of research utilisation among nurses and midwives working in public hospitals was studied. There was limited support for nurses’ and midwives’ experience of research utilisation. Nurses and midwives did not utilise research in their clinical practice intentionally. The knowledge, negative attitude towards research utilisation, lack of training, time mismanagement, and lack of motivation were the perceived barriers to research utilisation. Therefore, the promotion of adopting the research utilisation and training on the identified barriers are mandatory.

**Supplementary Information:**

The online version contains supplementary material available at 10.1186/s12978-021-01095-x.

## Background

Research utilisation in clinical decision-making practice is the use of best, valid, and currently available and relevant research findings in clinical and healthcare decision-making practice. It improves patient outcomes [1]. The knowledge, skill, experience of health service providers, and patient preference are basic for research utilisation (RU). Nurses and midwives who have higher educational status, and the experience of management and service provision can reduce barriers of research utilisation in clinical decision-making practice through collaborative decision-making, and good time management to improve quality healthcare [2].

A large amount of primary research and systematic review findings are produced continually in healthcare. If these findings are transferred to practice, the quality of healthcare can be improved [3, 4]. However, an increase in the bulk of available research findings does not automatically transfer into knowledge and practice to improve patient care and treatment [5]. It is rarely used for clinical decision-making practice. Nurses and midwives utilise experienced-based knowledge and their observations, colleague, and other collaborators for support in practice without considering best and current research findings [6].

Training of research for nurses and midwives who are master’s and Ph.D. holder is not common even in European countries like France to utilise research in clinical decision-making practice. Research is familiar to higher educational institutions. The limitation is that there are not yet any professional available which enable research utilisation in the healthcare and clinical decision- making practice [7]. The experience of research utilisation among nurses and midwives is perceived as a basic and pivotal part of unified professional nursing and midwife. Research utilisation is consequently viewed as contributing to the quality of healthcare and renewal of better patient outcomes [8], as well as the development of nursing education and leadership in clinical decision-making practice [9].

The study conducted in South Africa indicates that the mixed effort of local and international researchers with clinicians can create a culture of research utilisation within one country. It also shows that research utilisation in health services requires time and perseverance from international researchers together with readiness by local researchers to take and actively promote research utilisation [10]. It is suggested that knowledge translation should usually be up-to-date systematic reviews or syntheses of research findings. It mainly emphasizes investigating research utilisation methods capable of encouraging the exchange, transfer, diffusion, and distribution of evidence-based knowledge to practitioners and decision-makers in healthcare systems [11].

Research utilisation should contain solving complex problems that are basic in nursing and midwifery healthcare [12]. Nurses and midwives have to address the research and practice gaps through the insertion of research knowledge into clinical practice, i.e. an evidence-based practice (EBP). To fulfill this proposed role, nurses and midwives have to prepare their clinical expertise [13]. Studies suggest that research utilisation is intervened by the interplay between the individuals, the new knowledge, and the real context. There should be support to organize and use research in daily practice [14, 15]. In addition to this, the applicability of research findings should be locally evaluated and the results of the evaluation must be made actionable and usable, and adapted to the local situation [16–18]. However, nurses and midwives lack highly specialized clinical expertise and research leadership responsible to support new knowledge transfer from research to clinical decision-making practice with bedside clinicians [13].

Many barriers hamper the engagement of nurses and midwives in research utilisation [19, 20]. Comprehensive assessment of the experience of research utilisation can help for the development of appropriate strategies to reduce or get rid of barriers [21]. The use of research findings in daily clinical practice is crucial to bridge the continuing gap between research utilisation and clinical decision-making practice [22]. There is a scarcity of literature that examines the experience of nurses and midwives towards barriers and supporting factors for research utilisation in Ethiopia. Moreover, many barriers like lack of motivation and training on how to utilise research in healthcare were not studied. Therefore, this study was designed to explore nurses’ and midwives’ experience of research utilisation in public hospitals.

## Methods

### Study design and setting

A qualitative descriptive approach was employed from January 3 to June 28, 2020, to explore nurses’ and midwives’ experience of research utilisation in clinical decision-making practice within South Gondar Zone public hospitals. South Gondar Zone is one of the zones of Amhara Regional State in Ethiopia. The study included one general hospital (Debre Tabor) which serves about 2.3 million people and each of the seven primary hospitals (Mekane-Eyesus, Addis Zemen, Andabet, Ebinat, Nefas Mewucha, Tach Gayint, and Wogeda) serves more than 150,000 people. Debre Tabor General Hospital served as a teaching hospital of Debre Tabor University.

### Study participants

Twenty interviews (5 key informants and 15 interviewees), four FGDs (focus group discussions), and eight observations were conducted. The key informants included one medical doctor, one hospital manager, and three masters in emergency surgery and obstetrics. Each FGD consisted of eight to twelve participants. A checklist was employed to observe the availability of resources/materials used for research utilisation in the eight hospitals. A checklist was also employed to observe nurses’, and midwives’ roles, and the availability of resources/materials used for research utilisation in the clinical practice. A total of 67 participants were involved in the study. Nurses, midwives, doctors, and masters in the emergency surgeon and obstetric participated in the in-depth interview. Nurses and midwives participated in FGDs. Name of nurses, midwives, doctor, and masters in the emergency surgeon and obstetrics was coded for the participants of an in-depth interview (01–20), FGD1 (FGD1-01 to 12), FGD2 (FGD2-01 to 12), FGD3 (FGD3-01 to 12), FGD4 (FGD4-01 to 12) and observation (H01- H08) (see Additional file 1). A purposeful sampling technique was used to recruit participants for an in-depth interview, FGD, and observations.

### Data collection

An in-depth interview, FGD guides, and checklist for observation were developed by reviewing literature and feedback of experts in research utilisation. The interview and FGD guide were divided into four categories i.e. the experience of research utilisation, barriers of research utilisation, availability of resources/materials in the hospital for research utilisation, and the support for research utilisation. The in-depth interview and FGD guide and checklist for observation were prepared first in English then translated to Amharic and retranslated back to English for consistency. Four data collectors (research assistance) who had the educational status of master and Ph.D. with previous qualitative data collection experience were selected. They were trained to be familiar with the objective and the methodology of the research. Data of on-site research utilisation of nurses and midwives were collected and all hospitals in the zone were considered. Data were collected via FGD and face-to-face interview technique using semi-structured questionnaires. Good communication started with the greeting and the ground rule had been set before the FGD (focus group discussion) started. The interview duration was between 45 and 60 min, and the FGD duration was 90 min to 120 min.

Moreover, the data collectors and investigators were engaged in participatory observation using a checklist. The participants’ emotions and non-verbal communication were recorded as field notes. The interviews and FGDs were audio-recorded and then later transcribed for analysis. Saturation was determined when there were multiple overlapping responses across participants.

### Data processing and analysis

The in-depth interviews and FGDs were transcribed verbatim first in Amharic and then translated into English and retranslated back to Amharic to check for consistency. The transcripts were read repeatedly and checked independently by investigators for confirmation. Initially, data from observation, interview, and FGD were imported into NVivo 12 plus to manage and analyze the data using the Computer-Assisted Data Analysis Software Program (CAQDAS). The data were analyzed through thematic content analysis. First, a list of codes was created and described. Then after adding and defining the concept, themes were developed. The number of categories was reduced by” collapsing those that are similar or dissimilar into broader higher-order categories” [23]. Finally, the codes were ordered into different themes, and the main theme, themes, and subthemes were identified. Moreover, essential quotations were clustered. The quotations were used to elaborate on the context that affects the experience of participants and how the participants experienced the phenomena.

### Trustworthiness

The investigators, research facilitators, and nurses’ and midwives’ experts were invited to review the study’s findings and the right idea that represents their point of view was taken for the study to maintain credibility. Dependability was addressed by analyzing all the interview, FGD, and observation transcripts by at least two researchers with a third-checker to make ensure consistency across the data analysis process [24, 25]. Moreover, the investigators and research facilitators discussed the emerging themes from the dataset and resolve any different perspective by foraging consensus on interpretation [26]. The decision of transferability of the phenomenon to a new set of situations depends on the contextual information provided by the investigators. Thus, we expect that there is an affluent description that can support the reader to know the situation in this report.

## Results

A total of 67 participants were involved in the study.Thirty-eight participants were married and thirty of them were single.The participants’ age ranged from 24 to 55 years and their mean age was 30 years. Forty-six male and twenty-one female participants participated in the study. The participants’ work experience ranged from 8 months to 32 years and its mean was 7.5 years. Of the total participants, 37 (55.2%), 25 (37.3%), 5 (7.5%) were nurses, midwives and key informants respectively. Two MSc nurses, thirty-five BSc nurses, two MSc midwives and twenty-three BSc midwives participated in the study. In terms of participants’ position, four head nurses and four head midwives participated in the study. One medical director, one hospital manager, two quality healthcare coordinators, and three case managers also participated in the study.

The analysis of data from observation, FGD, and interview produced one main theme and three themes. The three themes were the participants’ belief in research utilisation, the limited support for research utilisation, and the perceived barriers of research utilisation.

### The non-intentional research utilisation

This is the main theme which included the definition of research utilisation. Research utilisation is defined as applying knowledge obtained from research in clinical practice. Non-intentional research utilisation is defined as the process of applying information and knowledge obtained from research findings in clinical and healthcare decision-making non-deliberately. This is described by one of our FGD participants as follows:

*“I understand research utilisation. It is using trusted research findings in healthcare practice. I know nurses and midwives who utilise research findings instead of hospital protocol in the healthcare practice. I use guidelines and hospital protocols to get knowledge and skills for my healthcare decision-making. I utilise research sometimes when I get trusted research findings. I don’t utilise it intentionally (FGD2-03)*.”

### Participants’ believe in research utilisation

Participants’ belief in research utilisation is defined as the participants’ trust to utilise research in clinical decision-making practice. The interviewees and FGD participants believed that they didn’t have to utilise research findings in healthcare and clinical practice, because it wasn’t approved by the responsible bodies. One of the interviewees described his belief in research utilisation as follows:

*“I did not utilise single primary research due to fear of patient harm and accountability, and clients might not get uniform health care through all health facilities. New research finding should be tested if it works in our setting. I do not utilise research for my decision if it is not approved by the responsible bodies. The responsible bodies or higher officials didn’t allow us to do so (19).”*

### The limited support for research utilisation

The theme ‘the limited support for research utilisation included the subthemes’ the supportive organization to utilise research, the NGOs’ and other stakeholders’ support to utilise research findings in clinical decision-making practice, and mentoring, supportive supervision, monitoring, and evaluation of RU. The support for nurses’ and midwives’ RU and the role of managers for supportive organizations affect the ability to utilise research.

### The organizations’ support for research utilisation

Nurses and midwives’ managers and ward heads understood that supportive organizational resources like electronic journals, work-based libraries, and access to research articles had an impact upon RU. Training, mentoring, giving time for research activities, and managers who embrace research were vital in research utilisation. However, managers didn’t facilitate supportive organizations to utilise research thinking that priority issues were more important than supporting research utilisation. This was described by one of the interviewees:

*“I know research utilisation needs special supports like training, mentoring... I don’t expect this kind of support for the nurses and midwives. It is impossible. I had a lot of else activities that had to be done. You give priority when you do your job. Urgent issues were very common in our day-to- day activities. There is no system and supporting the way of evidence-based practice in our hospital. It may be our future home take assignment (01)”.*

The lack of support decreased nurses’ and midwives’ confidence to utilise research in healthcare and clinical practice. There was a fear of accountability for patient harm due to the lack of support for nurses and midwives to utilise research in clinical practice. The FGD participant described his experience as follows:

*“My hospital has a protocol to control diabetic ketoacidosis. We use this protocol. I use one research finding that showed the treatment dose of insulin was based on the weight of the patient. Diabetic Mellitus was controlled immediately when I compared it with our hospital protocol. I did it by myself. It is not official. But our protocol does not use the treatment based on patient weight and it takes time to control ketoacidosis. We have fear in using research because of harmfulness to the patient. There should be trust to use it. Do you see here? I didn’t get any training. We do not have a computer. What do you mean? There should be a supporter to give us confidence in using research. We are dependent on hospital protocols and guidelines (FGD2-03)*.”

### NGOs’ and other stakeholders’ support for RU

The study showed that NGOs aren’t working in the database to help us access the best research findings. Participants didn’t get the support of both local and international organizations for research utilisation. There is no training considering research findings to utilise it in clinical and healthcare decision-making practice. One of the interviewees described the NGOs’ and other stakeholders’ support as follows:

“*It is very difficult to think about this. It is one of our challenges. How can we get research? It could be accessible in colleges and universities. NGOs do not work on this…Oh… there is nothing to support nurses and midwives to utilise research in clinical activities (03).”*

### The supportive supervision, monitoring, and evaluation of RU

The analysis of data from FGD and interview indicated that mentoring, and supportive supervision, control and evaluation were rarely implemented to change RU. There was no system to mentor and support research utilisation in clinical practice. One of the interviewees stated his experience:

*“I know that mentoring and supportive supervision can change RU. However, we don’t have any system to mentor the use of research in the clinical area. I don’t think that even higher officials had this idea of supportive supervisors and control to utilise research. I do not expect the activity of research in our hospital. Nurses and midwives may do this individually. Otherwise, research activity is limited to colleges and universities (05)”.*

### The perceived barriers to RU

The theme ‘the perceived barriers of RU’ involved the subthemes knowledge and skills, lack of resource and training, time mismanagement, and lack of motivation. The analysis of data from the interview, FGD, field notes, and observation identified the barriers to research utilisation.

### Knowledge and skills

FGD and interview participants had awareness of their knowledge gap to use knowledge and information from research findings in clinical and healthcare decision-making practice. Further, these nurses and midwives felt as they had no skills to utilise evidence like quality research. One of the key informants of the study describes this as follows:

*“We couldn’t read research articles due to the knowledge gap to differentiate the best and current research finding. If we update ourselves by research knowledge, it is possible to provide quality health care. This is important for health service providers, patients, and hospital (13).”*

### Time mismanagement

Data analysis indicated that time mismanagement was the barrier to RU. One of the interviewees described this barrier as follows:

“*There is a very great workload. I cannot read anything during working hours. I cannot read even at home. After working hours, I go home and I want to sleep due to tiredness. It is impossible to go to the library and read an article and it is impossible to search journals due to lack of time. I have to get time either during working hours or at home to search and read journals related to our health service (FGD3-09).”*

### Lack of resources and training

The data from FGD, interview, and observation indicated that participants did not utilise research due to the inaccessibility of research articles and training on how to utilise it. One of the key informants described the condition:

*“We cannot use single research in healthcare practice. It is difficult to get a systematic review and meta-analysis of research findings. Our hospital has no library and computers. Further, most nurses and midwives didn’t get training to utilise research findings in the healthcare practice (20).”*

The analysis of data from observation indicated that there were no libraries and computers in the majority of hospitals. Most nurses and midwives didn’t have access to articles to transfer knowledge of research findings into clinical decision-making practice. Most of the procedures were performed without referring best and currently available research findings (H08).

### Lack of motivation

The majority of FGD and interview participants claimed that motivation is one of the issues of research utilisation. They describe personal encouragement and motivation to utilise research during health service provision. It is impossible to change the existing traditional practice without personal drive or motivation. The study participants agreed that lack of motivation was one of the barriers to research utilisation. This was discussed by one of the FGD s participants:

“*I do not utilise research for my clinical practice. I always do the same procedure and my colleagues too… I feel frustrated. Nothing is encouraging. Sorry to say this. I thought that there is no need to use research for these repeated activities in the clinical setting (FGD3-06).*”

Nurses and midwives blamed that managers did not treat the hospital staff equally. Managers’ mishandling decreases nurses’ and midwives’ interest and motivation to do their job. One of the participants stated that unequal respect of hospital workers decreases motivation to use journals for clinical practice.

“*The managers do not respect hospital workers equally. It becomes a culture. There is a problem with the hierarchy. From the top, there are senior doctors. At the bottom level, there are janitors and guards. The attitude of managers to respect their workers decreases from top to bottom. Even if, nurses and midwives get evidence from research and try to utilise it, nobody accepts us. How do you feel this kind of demoralization? Ohm… I cannot explain it (FGD3-02).”*

## Discussion

The support for research utilisation in clinical and healthcare decision-making practice is crucial. However, there was inadequate support for nurses and midwives to utilise research in healthcare practice. Barriers to research utilisation were the main concern of nurses’ and midwives’ experience to utilise research in clinical decision-making.

This study indicated that nurses and midwives did not work intentionally using research findings by themselves. Participants believed that primary research articles were not trusted to utilise in clinical decision-making. Some of the participants of this study are concerned that the utilisation of research findings in clinical decision-making increases accountability for patient harm and restrains equity health services due to nurses’ and midwives’ utilisation of different research findings in healthcare. Therefore, they were not trusted to utilise research findings. This could be because of a lack of training and promotion to utilise research findings in clinical decision-making. As far as our searching for similar studies, no other studies could have reported this finding. This finding showed that the responsible bodies should test the research findings and there should be standards to utilise research. This finding agreed with the study which indicated that understanding the strength and limitation of the research gives the confidence to utilise research [27]. This finding is also in line with other studies which indicated that giving value for research and development of standard measurement of research findings were important to utilise research in healthcare [28, 29].

Our study found that nurses’ and midwives’ confidence to utilise research was recognized as the result of the organizations’ support for research utilisation. There was participants’ fear to use research where there was the perception of a lack of supportive organization. Similar to this study finding, other studies found that organizational support to get advanced training, resources, and mentors were vital to utilise research in clinical decision-making [30–33]. This could be due to the decreasing of barriers of research utilisation through the supportive organization to alleviate the tension of nurses and midwives during their health care practice. Resources could also be accessed easily to utilise research in the health care decision-making, where strong supportive organization to research utilisation existed.

This study presented that the barriers of research utilisation were time mismanagement, lack of knowledge, negative attitude, lack of motivation, lack of support, and lack of resources. These barriers could be categorized under individual and institution level barriers. Studies conducted in Canada, Ghana, Germany, Iran, China, and Jordan presented these barriers of research utilisation [19, 30, 34–37]. However, the perceived causes of these barriers to utilise research vary in these studies. For example, our study finding revealed that the nurses and midwives weren’t motivated due to managers’ favor for doctors than nurses and midwives. Otherwise, the study conducted in the USA revealed that the lack of motivation was common among nurses working for long years in one health facility which resulted in the loss of interest due to the length of time between formal academic training and current employment [31]. Denmark’s study also revealed that lack of motivation of nurses presented and the perceived causes of lack of motivation were failed to utilise research in the healthcare decision-making due to nobody took action on the planned activities [38]. Whereas, our study also presented that the participants perceived that they were doing the same procedure and they were frustrated in doing the same thing. Thus, they lost their interest to utilise research in clinical practice.

In this study, participants were seen when they were performing their procedure traditionally. They had no support to utilise research that can add nurses’ and midwives’ confidence. They did not utilise research in clinical practice due to fear of accountability for patient harm. They didn’t disclose even their research utilisation in clinical decision-making. The lack of support in this study was different from that of the study conducted in Denmark. The lack of support presented in the study of Denmark was non-formalized at the organizational level and not led by management. However, participants in the study of Denmark had shared new research findings, and a consensus decision was taken to utilise the new research findings [38]. The possible reason for this difference could be due to better promotion of adopting to utilise research findings in Denmark’s study. Our study also indicated that managers had priority of administrative issues rather than supporting nurses and midwives to utilise research. Managers didn’t give attention to research utilisation in the health care practice due to overlap of activities and they didn’t consider research utilisation as a priority activity. This masks the supportive organization for research utilisation. This kind of mask to support research utilisation due to task overlap was also reported in the study conducted in the UK [39].

One of the outcomes identified as a subtheme was “lack of resources and training.” The participants couldn’t get systematic review and meta-analysis research findings and guidelines to utilise research findings. They had a knowledge gap. Even if they get these journals, they didn’t utilise the findings due to lack of training. They argued that the utilisation of primary research findings in clinical practice was not important. They described that a single primary research finding may work for that particular study area. It may not be used for all settings. As far as our search for other similar studies, no other studies reported this argument.

## The strengths and limitations

The study’s major strength is that the investigators, research facilitators, and nurse and midwife experts reviewed the findings of this study and it is presented at the meeting. The risk of bias was restricted by ensuring privacy for the interviewees and a quiet room to conduct FGD. This study addressed that research utilisation in this study is typically underpinned through handling supporting factors for research utilisation, reducing perceived barriers of research utilisation, and improving the experience of research utilisation.

The first limitation of this study was the possibility for social desirability bias as the study was conducted using interview and FGD methods, while nurses and midwives were working in the hospitals. Moreover, the response of the participants might be inflated or underestimated due to individuals with some interests. Second, this study was conducted in hospitals where a more advanced human resource dynamic, quality medical service, and well-organized structure were available. Hence, transferability is difficult for health centers and health posts.

## Conclusions

The nurses’ and midwives’ experience of research utilisation indicated that there was limited support for nurses and midwives to utilise research in clinical decision-making. Nurses and midwives did not utilise research in their clinical practice intentionally. The findings of this study have identified that knowledge, negative attitude towards research utilisation, lack of training, time mismanagement, and lack of motivation were the perceived barriers of research utilisation. Therefore, the promotion of adopting research utilisation, and training on the identified barriers are mandatory (Table 1).

**Table 1:** Summary of themes for nurses’ and midwives’ experience of research utilisation in clinical decision-making practice within public hospitals, Ethiopia, 2020

Main theme	The non-intentional research utilisation		
Themes	The participants’ believe in research utilisation	The limited support for research utilisation	The perceived barriers of research utilisation
Subthemes	Participants’ trust	The organizations’ support	Knowledge and skills
		NGOs’ and other stakeholders’ support	Lack of resource and training
		The supportive supervision, monitoring, and evaluation	Time mismanagement
			Lack of motivation

## Supplementary Information

**Additional file 1.** Interviews.

## Data Availability

The datasets used and /or analyzed during the current study are available from the corresponding author on reasonable request.

## Abbreviations

RU: Research Utilisation
FGD: Focus Group Discussion
