# Supplementary material for: Research utilisation in clinical practice: the experience of nurses and midwives working in public hospitals
Source: Reprod Health. 2021 Mar 15;18:62. doi: 10.1186/s12978-021-01095-x (PMC7962333; doi:10.1186/s12978-021-01095-x)
Supplement: Supplementary file 1 — Additional file 1. Interviews. [file 12978_2021_1095_MOESM1_ESM.docx]

**Interviews**

1. **How do you believe research utilisation in your hospital? How do you perceive research utilisation?**

Response of interviewee (key informant): 01

I believe nurses and midwives have perception of research utilization because of their many endevours to improve their performance. Most of nurses and midwives are young. We are interested to improve our knowledge. I think nurses and midwives have good attitude but we cannot measure knowledge during observation.

1. **How do you think the importance of research utilisation? What are important articles used in clinical practice?**

Response of interviewee (key informant): 01

Research utilization is important. There are some guidelines in this hospital. There is also training support. Mostly, we use sharing of experience and evidence among nurses and midwives for clinical practice. We do not use research articles. There are not any updated materials like hospital protocols, guidelines and research articles in our hospitals.

1. **What are the reasons that you do not utilise research for your clinical decision-making practice?**

Response of interviewee (key informant): 01

Our hospital library is small. It has no space to read books and research articles that can help us in clinical practice. The library is not comfortable .In addition to this; there is shortage of time due to work load of nurses and midwives. The manager and ward heads do not supervise and control nurses and midwives’ research utilization.

1. **How the supports for research utilisation important?**

Response of interviewee (key informant): 01

I know research utilisation needs special supports like training, mentoring... I don’t expect this kind of support for the nurses and midwives. It is impossible. I had a lot of else activities that had to be done. You give priority when you do your job. Urgent issues were very common in our day-to- day activities. There is no system and supporting the way of evidence-based practice in our hospital. It may be our future home take assignment.

**1. How do you believe research utilisation in your hospital? How do you perceive research utilisation?**

Response of interviewee (key informant): 02

I believe that research is important to give quality healthcare

**2. How do you think the importance of research utilisation? What are important articles used in clinical practice?**

Response of interviewee (key informant): 02

In any health institution, providing health care service based on research findings is important. I think the community that gets health care service from research is appropriate. The community need of health care service is increasing. Equivalent health service for increased need of community for health care is impossible without research utilization. Health care service can not satisfy the community without research utilization. The success of health care is the result of research utilization.

**3. What are the reasons that you do not utilise research for your clinical decision-making practice?**

Response of interviewee (key informant): 02

Nurses and midwives do not implement use research because of negligence, lack of awareness, lack of motivation, social and economic reason.

**4. How the supports for research utilisation important?**

Response of interviewee (key informant): 02

We don’t have onsite training programme for newly engaged staff. There aren’t newly launched standards of nursing and midwifery care. We provide health services for the identified gaps without training. There is morning session including doctors on Monday and Friday. During morning session the staffs share knowledge from research sometimes.

**1. How do you believe research utilisation in your hospital? How do you perceive research utilisation?**

Response of interviewee (key informant): 03

I believe that nurses’ and midwives’ use of research articles is through evaluation and observation. When we see vaccine service, first nurses or midwives get training, then they evaluate the research findings. If there is gap, we consider that there is no further reason to use research knowledge in clinical practice.

**2. How do you think the importance of research utilisation? What are important articles used in clinical practice?**

Response of interviewee (key informant): 03

We know nurses and midwives’ research utilization through evaluation and observation. When we see vaccine service, first nurse or midwife get training, then we evaluate performance. If there is gap, we consider that there is no research utilization.

I think nurses and midwives use research may be sometimes. Lack of research utilization is not good. It causes poor quality health care. The use of research for implementation of evidence based practice is important. Because these research articles could be updated source of knowledge.

Research utilization is very important. It improves patient care. However I don’t use it. Most of the time nurses and midwives use national guidelines for implementation of evidence based practice. But we do not use it always. We cannot get research articles in our hospital and we cannot use it. We can get from internet but there is not internet access in our hospital. We use senior nurses and midwives as a source of knowledge and skills through sharing of their experience.

**3. What are the reasons that you do not utilise research for your clinical decision-making practice?**

Response of interviewee (key informant): 03

There are factors like internet access, lack of library, lack of time etc that hinder our use of research findings. The other is lack of nurses’ and midwives’ knowledge.

**4. How the supports for research utilisation important?**

Response of interviewee (key informant): 03

It is very difficult to think about this. It is one of our challenges. How can we get research? It could be accessible in colleges and universities. NGOs do not work on this…Oh… there is nothing to support nurses and midwives to utilise research in clinical activities.

**1. How do you believe research utilisation in your hospital? How do you perceive research utilisation?**

Response of interviewee (key informant): 04

I believe that research utilization increase quality health care if we use it properly. Previously, knowledge from research was not known. But research utilization has been included in reform since 2012. There are guidelines for research utilization. There are 20 chapters for nurses and midwives in the reform. According to the reform nurses and midwives can prepare their own guidelines and they are autonomous to have their own policies. Nurses and midwives have 10 standards and operational standards. These standards are prepared and set in all departments without considering knowledge from research.

**2. How do you think the importance of research utilisation? What are important articles used in clinical practice?**

Response of interviewee (key informant): 04

Research utilization is important for quality healthcare service and patient satisfaction. If patients are not satisfied, they find getting good service to other health institution. Therefore research utilization is mandatory.

There is ministry of health direction which allows the use of all trusted evidences for nurses’ and midwives’ research utilization. Important research findings can be reported to hospital management committee and used as benchmark even for clustered hospitals.

**3. What are the reasons that you do not utilise research for your clinical decision-making practice?**

Response of interviewee (key informant): 04

Nurses and midwives do not use research as a source of knowledge because of lack of access of materials, nurses and midwives stick to traditional practice, negligence, lack of incentive, and lack of managerial support. Lack of material access is one of the challenges in our hospital. The hospital is established recently. We buy materials for fetching water and internet card to down load books, guideline and others by taking money from our pocket. There is not bed and laundry for washing bed-sheet. IF more patients come for operation, we do nothing. These all challenges have impact to use research finding as source of knowledge

**4. How the supports for research utilisation important?**

Response of interviewee (key informant): 04

There is established group for team management. The nursing team management prepares guidelines. There is also selected supervision committee. I am the head of nursing supervisors and we supervise nurses and midwives according to the schedule using check list. Based on the identified gaps during supervision, here is supportive intervention

Non-governmental organizations are the only stakeholder to support the use of training manuals and guidelines. However I did nothing to communicate with stalk holder about the use of research findings.

**1. How do you believe research utilisation in your hospital? How do you perceive research utilisation?**

Response of interviewee: 05

Research utilization is very useful. As you know .Medicine is updated every time. Evidence that we use today may not work for tomorrow. I believe that clinical decision based research finding is vital. When a patient comes to hospital, he can get quality health care.

**2. How do you think the importance of research utilisation? What are important articles used in clinical practice?**

Response of interviewee: 05

Nurses and midwives should use research articles. This is very important for knowledge and skill of nurses and midwives. Short term and long trainings are also important. There should be knowledge transformation.

Nurses and midwives should always discuss about new cases they get during their clinical practice. Hospital should have morning session forum. Nurses and midwives should follow attentively.

Some activities of medicine do not have guidelines. Few procedures have national guidelines. It is impossible to address all medical activities using guidelines. When I see practically, there are communicable disease control, maternal and child treatment guidelines. There are not non-communicable disease guidelines. Nurses and midwives use research findings instead of national guidelines. Nurses and midwives can use research to improve their knowledge and skill. There is not any prohibition to use evidences. But our research utilization should be safe for the patient.

**3. What are the reasons that you do not utilise research for your clinical decision-making practice?**

Response of interviewee: 05

There is no human resource model 15 for district hospitals. There is no library personnel. The hospital has no library. Nurses and midwives use internet for social media and self entertainment instead of using research finding as a source of knowledge.

**4. How the supports for research utilisation important?**

Response of interviewee: 05

I know that mentoring and supportive supervision can change the use of research knowledge. However, we don’t have any system to mentor the use of research in the clinical area. I don’t think that even higher officials had this idea of supportive supervisors and control to utilise research. I do not expect the activity of research in our hospital. Nurses and midwives may do this individually. Otherwise, research activity is limited to colleges and universities

**1. How do you believe research utilisation in your hospital? How do you perceive research utilisation?**

Response of interviewee: 06

I believe that research utilization is important. It improves patient outcomes. However I do have question how to use research findings.

**2. How do you think the importance of research utilisation? What are important articles used in clinical practice?**

Response of interviewee: 06

I think the importance is clear. It is for better health service. Research findings are main source of knowledge. No one deny this.

**3. What are the reasons that you do not utilise research for your clinical decision-making practice?**

Response of interviewee: 06

I do not use research findings for clinical decision-making practice because of lack of internet access, lack of knowledge, negligence, may be lack of attitude and lack of understanding about research articles.

**4. How the supports for research utilisation important?**

Response of interviewee: 06

Our hospital has committee to evaluate the use of guidelines and hospital protocol. The committee evaluates the availability of guidelines and hospital protocols quarterly. The evaluation identified that the use of guidelines and hospital protocols is low in some wards and good in the other wards. However, there is no evaluation and support to use research findings in clinical practice

**1. How do you believe research utilisation in your hospital? How do you perceive research utilisation?**

Response of interviewee: 07

I believe that the use of research is to judge trustfulness of research by using our previous knowledge and scientific explanatation. It is way of sharing new knowledge among health service providers.

**2. How do you think the importance of research utilisation? What are important articles used in clinical practice?**

Response of interviewee: 07

The use of research as a source of knowledge is a project to solve the problems. There are few nurses and midwives who use research article in clinical practice.

**3. What are the reasons that you do not utilise research for your clinical decision-making practice?**

Response of interviewee: 07

Nurses and midwives know the importance of research findings as a knowledge source. However, there are challenges to use for clinical decision-making practice. The first one is work overload. Health care is becoming forceful activity. Nurses and midwives become tired due work overload. When they are tired, they do only to complete the patient card. Nurses and midwives do not use research for source of knowledge and skill because of lack of commitment and lack of experience to research articles.

**4. How do you see the supports for research utilisation in your hospital?**

Response of interviewee: 07

There is no system of supportive supervision and controls of research utilization among nurses and midwives in our hospital. However, I only tried to share confidence on how to consult cases for seniors. I shared my knowledge and skill of family planning for my colleagues after I had got training. There was no support for research utilization in our hospital\

**1. How do you believe research utilisation in your hospital? How do you perceive research utilisation?**

Response of interviewee: 08

I believe that the use of research is nurses’ and midwives’ acceptance to use knowledge obtained from research findings. Otherwise, we use our experience without using knowledge from research.

**2. How do you think the importance of research utilisation? What are important articles used in clinical practice?**

Response of interviewee: 08

Research findings are very important source of knowledge. However, most of health care managements are based on seniors. Some of experienced senior nurses and midwives are good and others are not good to use knowledge from research. Nurses and midwives are far from research finding, unless academic staffs use it. Therefore, nurses and midwives should update their knowledge through reading research articles.

**3. What are the reasons that you do not utilise research for your clinical decision-making practice?**

Response of interviewee: 08

Some patients know that public health workers do not read evidences like research articles. In high-income countries there is examination to get professional license after every two year. There is no this kind of licenser exam in our country that encourage nurses and midwives to use different evidences for their source of knowledge

**4. How do you see the supports for research utilisation in your hospital?**

Response of interviewee: 08

There are not stakeholders that support nurses and midwives for research utilization. I tried my best individually. I did prepare notes, power point and download articles and print these evidences to place in each ward so that nurses use these evidences to update our knowledge and skills.

**1. How do you believe research utilisation in your hospital? How do you perceive research utilisation?**

Response of interviewee: 09

I believe that the use of knowledge from research determine better health service especially for antenatal care ( ANC), family planning and other services. Research utilization is mandatory for patient satisfaction and it helps nurses and midwives to provide quality health care without difficulty. I don’t think nurses and midwives use research findings in clinical practice.

**2. How do you think the importance of research utilisation? What are important articles used in clinical practice?**

Response of interviewee: 09

Research utilization is means to upgrade knowledge of professionals. For example, nurses and midwives provide malaria management service using knowledge from research findings like guideline. If they have the knowledge, they can provide the service with confidence. I tried to get guidelines and manuals when I went to zones and health bureau for meeting. In addition to this, when nurses and midwives got traing and meeting, they share knowledge for all the staffs. Moreover, if we can use trusted research findings, it is additional source of knowledge.

**3. What are the reasons that you do not utilise research for your clinical decision-making practice?**

Response of interviewee: 09

There is work overload in outpatient department. Nurses and midwives do not write even patient assessment and report. Therefore, it is better to decrease patients per provider to implement to use research. We also did not create awareness of nurses and midwives to use research in clinical practice.

Nurses and midwives’ negligence is one of the problems to use research findings. This negligence is because of attitude. There could be lack of knowledge and skill .It depends on individual difference. I do not think there could be significant gap difference among nurses and midwives to intervene through training and morning session. Management support is also a factor for research utilization. Nurses and midwives are dissatisfaction because of lack of inceptives that leads to negligence. Training program was withheld by manager when nurses and midwives got training opportunity in our hospital. This could be cause for negligence.

**4. How do you see the supports for research utilisation in your hospital?**

Response of interviewee: 09

We have supportive follow up during morning session and other meeting. During mentoring, nurses and midwives fill their gaps. However, there is no planned supportive supervision and controls of research utilization in this hospital.

**1. How do you believe research utilisation in your hospital? How do you perceive research utilisation?**

Interviewee response: 10

I believe that it is use of approved research findings in clinical area. However, I don’t use research findings to improve my knowledge of clinical practice. I work in gynecology ward. We have guidelines. Most of the time, I use abortion guidelines. Many of nurses and midwives came from other hospital and they have work experience. We share experience. Most of gynecology procedures do not have guidelines. We use seniors as a source of knowledge and skills.

**2. How do you think the importance of research utilisation? What are important articles used in clinical practice?**

Interviewee response: 10

I do have no doubt about the importance of research use. I think that use of research finding in clinical practice is source of knowledge, skill and attitude. Nurses and midwives are updating themselves through improving their level of education and research knowledge and skill.

**3. What are the reasons that you do not utilise research for your clinical decision-making practice?**

Interviewee response: 10

Nurses and midwives do not implement evidence based practice because of shortage of time due to work overload, lack of access of sources of evidences, lack of updated evidences, lack of library, long distance of home from hospital, lack of internet access in the ward. You see this ward. I cannot go distance to get internet from this ward due to workload. There is service of bus for nurses and midwives. Their home is far from this hospital. It takes time to reach home.

**4. How do you see the supports for research utilisation in your hospital?**

Interviewee response: 10

Supporting for research utilization is one of our main concerns. There are no managers and stakeholders encouraging the use of research as a source of knowledge and skills. However, when nurses and midwives get manuals and training, they share the evidence, knowledge and skill for those who do not get the training. We place guidelines and manuals in the ward. There is sharing of evidences among nurses and midwives during meeting. We also have seminar presentation and morning session discussion in our hospital including our ward.

**1. How do you believe research utilisation in your hospital? How do you perceive research utilisation?**

Interviewee response: 11

I believe that most of nurses and midwives have no experience to use knowledge from research in clinical practice. I do not think that all experienced nurses and midwives use research findings. We usually use nurses and midwives’ experience in clinical practice. We share knowledge and skill from their experience for implementation of evidence based practice.

**2. How do you think the importance of research utilisation? What are important articles used in clinical practice?**

Interviewee response: 11

It is good to use research. There should be responsible body to facilitate the use of research articles. All hospital wards should give services for many patients based on best available evidences’ This is research. There are many wards in this hospital and all nurses and midwives in these wards should have access to research articles.

**3. What are the reasons that you do not utilise research for your clinical decision-making practice?**

Interviewee response: 11

I have not supported nurses and midwives for research utilization. There is selected case presentation from each ward and there is sharing of experiences during case presentation. We have supportive supervision and controls in this session. However, there is not planned supportive supervision and control of nurses’ and midwives’ use of research findings in this hospital. We do not use it because of workload and lack of material access like internet. I think there is not lack of knowledge, skill and attitude in our hospital.

**4. How do you see the supports for research utilisation in your hospital?**

Interviewee response: 11

Supporting of nurses and midwives for research utilization is good in this hospital. I have not communicated to stakeholders and NGOs about the use of research in our practical area.

**1. How do you believe research utilisation in your hospital? How do you perceive research utilisation?**

Interviewee response: 12

I believe that most of midwives in this hospital are newly employed. They were recently graduated from colleges and universities. They have knowledge to use research in clinical decision. In addition to this, they share experience with experienced nurses and midwives especially experience of activities of operation room scrub nursing and waiting nurse services.

**2. How do you think the importance of research utilisation? What are important articles used in clinical practice?**

Interviewee response: 12

I know the importance of knowledge from research. Unfortunately, We do not have the access of research. Regarding to guidelines, there is magnesium sulphate protocol which is posted in delivery ward. We use this when we administer magnesium sulphate. There is also protocol for retroviral infection (RVI) patients. Newly engaged nurses and midwives share experience of neonatal resuscitation procedures from residences and senior midwives. Previously, there was computer desk top in our ward. We had soft copies of books, guidelines and other evidences on the desk top. But there are not any article and guidelines in our ward.

**3. What are the reasons that you do not utilise research for your clinical decision-making practice?**

Interviewee response: 12

There is internet access even in our ward. Some nurses and midwives forget their practice because of wrong use of internet like chating face book and following social media instead of using internet for searching best evidence in clinical practice. Nurses and midwives should use internet for the benefit of patients.

We work all the day without rest and we become tired. When we go home, we want to sleep because of tiredness. Nurses and midwives are not motivated to read the different evidences like books guidelines, research articles and other evidences. When nurses and midwives get uncommon cases, they use their mobile to get evidences from internet. But nurses and midwives do not use library to read and refer books. They have not plan to use research in clinical practice.

**4. How do you see the supports for research utilisation in your hospital?**

Interviewee response: 12

I have not got any stake holders working in research utilization. I have no authority to communicate with non-governmental organization. I did nothing about research utilization in clinical practice. I communicate to my senior heads about in-service training.

**1. How do you believe research utilisation in your hospital? How do you perceive research utilisation?**

Interviewee response (key informant): 13

I have trust to use knowledge from research. The problem is how to use it. I know that research studies are input of community service. Regarding to national guidelines, there are launched activities. These evidences are important to keep quality health care. There are many standards in our hospital currently. These are Ethiopian implementation guidelines, clean and safe hospital standard and other standards. Our hospital uses these guidelines and standards strongly. However, it misses the use of research in clinical practice

**2. How do you think the importance of research utilisation? What are important articles used in clinical practice?**

Interviewee response (key informant): 13

The use of research in clinical practice is better to improve skill gap and discussion among nurses create awareness of using research. Empowering nurses and midwives is mandatory to use research.

**3. What are the barriers that you do not utilise research for your clinical decision-making practice?**

Interviewee response (key informant): 13

We couldn’t read research articles due to the knowledge gap to differentiate the best and current research finding. If we update ourselves by research knowledge, it is possible to provide quality health care. This is important for health service providers, patients, and hospital

**4. How do you see the supports for research utilisation in your hospital?**

Interviewee response (key informant): 13

There are many stake holders in our hospitals. I am one of stakeholders as quality unit leader. There are team-leaders in each ward. There are also non-governmental organizations. These organizations prepare dash board. We it use to assess the progress of quality healthcare. However, there is no supportive assessment related to research utilization.

**1. How do you believe research utilisation in your hospital? How do you perceive research utilisation?**

Interviewee response: 14

I believe that nurse and midwives should use knowledge from research trustfully. I am not clear about evidences used for clinical decision in clinical practice. I know some of the evidences like hospital protocols, national guidelines, standard books and research articles. I do not know that research studies used as evidences for implementation of evidence based practice.

**2. How do you think the importance of research utilisation? What are important articles used in clinical practice?**

Interviewee response: 14

Research utilization minimizes mistakes. It improves quality health care service. However, we do not use research studies during clinical decision-making practice.

**3. What are the barriers that you do not utilise research for your clinical decision-making practice?**

Interviewee response: 14

Nurses and midwives do not use research because of shortage of materials like lack of library in our hospital, lack of experienced nurses and midwives, lack of good management system and lack of internet access.

**4. How do you see the supports for research utilisation in your hospital?**

Interviewee response: 14

Matrons are not task holders. However, if there is commitment, they can communicate with hospital manager and they can avail trusted guidelines that include knowledge from research findings. The chief manager and medical director haven’t met with nurses and midwives to discuss about research utilization.

**1. How do you believe research utilisation in your hospital? How do you perceive research utilisation?**

Interviewee response: 15

It is good to use trusted knowledge from research. I know the habit of research utilizati is low in our hospital and most nurses and midwives use national guidelines. There is gap of using other references like research articles and books.

**2. How do you think the importance of research utilisation? What are important articles used in clinical practice?**

Interviewee response: 15

Research utilization is vital. It is better to be obligation of nurses and midwives to use research knowledge in clinical practice. This is improves civil service. We cannot provide quality health service without knowledge and skills. We have to see this as our routine activities

**3. What are the barriers that you do not utilise research for your clinical decision-making practice?**

Interviewee response: 15

We have sought staff motivation. Based on this, there are nurses and midwives who have good motivation. There are also nurses and midwives who do not have good motivation. The other one is lack of internet access. Due to lack of internet access, nurses and midwives have difficulty to get faithful research findings.

**4. How do you see the supports for research utilisation in your hospital?**

Interviewee response: 15

There are not stakeholders working on research utilization in our hospital. There are not evidences distributed by health institution. We get guidelines during training only. In this case, non-governmental organizations are participated in this regard. Government hospitals also support training. Ministry of health and health bureau do not distribute guidelines. However, we do not have means to get research article except individual endevours.

**1. How do you believe research utilisation in your hospital? How do you perceive research utilisation?**

Interviewee response: 16

I have the trust to apply research knowledge. I think there should be orientation and training of nurses and midwives during meeting on how to use research findings. In addition to this, there is orientation about nursing standards and distributions of guidelines to each class. We have to evaluate quality service based on evidences and there should be action plan based on identified gaps.

**2. How do you think the importance of research utilisation? What are important articles used in clinical practice?**

Interviewee response: 16

I know it is important. Nurses and midwives get evidences like research articles and guidelines from internet. We also use books and our colleagues as a source of knowledge and skills. Most of nurses and midwives use guidelines for implementation of evidence based practice. Some nurses and midwives may use research articles for implementation of evidence based practice.

**3. What are the barriers that you do not utilise research for your clinical decision-making practice?**

Interviewee response: 16

We do not implement knowledge obtained from research findings because of work overload, lack of trust, lack of getting research articles, lack of awarnes, shortage of updated guidelines, lack of motivation, lack of skill, shortage of in-service training and we did not develop our skill in skill lab demonstration when we were in colleges and universities. There is also lack of research articles and internet access in our hospital.

**4. How do you see the supports for research utilisation in your hospital?**

Interviewee response: 16

There are stakeholders like quality unit officer in our hospital. In addition to this, ministry of health, health bureau, and non-governmental organization are stakeholders. As far as I know, there are not stakeholders that support the use of research in clinical practice. I know that I have raised question about this issues during staff meeting. The hospital manager is the main responsible body for this concern.

**1. How do you believe research utilisation in your hospital? How do you perceive research utilisation?**

Interviewee response: 17

I believe that nurses and midwives should current knowledge of research findings. There are procedures that have no guidelines. I understand that nurses and midwives use their knowledge that they get from colleges and universities for these procedures. We should also use latest research findings.

**2. How do you think the importance of research utilisation? What are important articles used in clinical practice?**

Interviewee response: 17

I understand that research is useful to serve the community. Drug is ordered for the patient according to standard guidelines. We can get research findings about the drug from internet. We can find getting updated evidences through internet.

Nurses and midwives perform their professional activities according to their job description. They also participate in management activities. They use national guidelines and standards to develop their knowledge, skill and attitude. There are hospital protocols and policies prepared in the hospital. In addition to this, nurses and midwives get training to develop their knowledge and skill. Otherwise, there is gap to research articles in clinical practice.

**3. What are the barriers that you do not utilise research for your clinical decision-making practice?**

Interviewee response: 17

The lack of internet access, shortage of getting updated guidelines, lack of awareness of nurses and midwives, lack of attention of higher officials or decision makers, lack of motivation of nurses and midwives, shortage of time, lack of library and lack of attitude prevent our use of research findings in clinical areas.

One cannot use any evidence unless approved by concerned bodies in the hospital. One can use for his general knowledge. However, he cannot use knowledge from research without the knowledge of hospital committee.

**4. How do you see the supports for research utilisation in your hospital?**

Interviewee response: 17

There are no stakeholders like decision makers, hospital management committees, health bureau, ministry of health, non-governmental organizations and others that support research utilization,

**1. How do you believe research utilisation in your hospital? How do you perceive research utilisation?**

Interviewee response: 18

We believe in nurses’ and midwives’ knowledge of research utilization. However, we do not use knowledge from research findings in clinical practice. This indicates that knowledge is different from practice. We may not use all research articles for clinical decision-making practice.

**2. How do you think the importance of research utilisation? What are important articles used in clinical practice?**

Interviewee response: 18

Research utilization is very important. First of all, nurses and midwives have got knowledge from their school. In addition to this, we develop our knowledge and skill using guidelines, books, and research articles. We get newly updated evidences through in-service training. Nurses and midwives also get new updated evidence during morning session.

**3. What are the barriers that you do not utilise research for your clinical decision-making practice?**

Interviewee response: 18

Nurses and midwives do not use research because of lack of access of evidences, negligence, lack of knowledge, lack of updated evidences, lack of attitude and skill and work overload. For example, we have to provide antenatal care service for 30 pregnant women daily but one midwife provide antenatal care service for around 80 pregnant women daily on average.

Nurses and midwives inclined to accept horizontal sharing of knowledge from research findings instead of vertical sharing of knowledge. We do not believe sharing of knowledge among colleagues of similar professions. However, our colleague may not know more than what I know.

**4. How do you see the supports for research utilisation in your hospital?**

Interviewee response: 18

There is no supportive organization. There are no stake holders that can support research utilization in clinical practice. Matrons, medical directors, case managers, chief clinical officers, hospital manager, health bureau, ministry of health and non-governmental organizations are not concerned about yhe use of research in clinical practice.

**1. How do you believe research utilisation in your hospital? How do you perceive research utilisation?**

Interviewee response: 19

We can use trusted research knowledge in clinical practice. We have guidelines for each ward and case team. Nurses and midwives can read these evidences to use in clinical decision. But they do not use research articles in clinical practice.

**2. How do you think the importance of research utilisation? What are important articles used in clinical practice?**

Interviewee response: 19

Nurses and midwives know the importance of research article hospital protocols, guidelines and training manuals in clinical decision-making practice. But we do not have hard copies of research findings. I think nurses and midwives may use research articles to get updated information.

**3. What are the barriers that you do not utilise research for your clinical decision-making practice?**

Interviewee response: 19

I did not utilise single primary research due to fear of patient harm and accountability, and clients might not get uniform health care through all health facilities. New research finding should be tested if it works in our setting. I do not utilise research for my decision if it is not approved by the responsible bodies. The responsible bodies or higher officials didn’t allow us to do so

**4. How do you see the supports for research utilisation in your hospital?**

Interviewee response: 19

Higher officials, case managers, zone department, health bureau, ministry of health and nongovernmental organizations are not involved in the use of knowledge from research findings in clinical practice. There are no non-governmental organizations that support research utilization as they do in training, supporting of access to training manuals and guidelines.

**1. How do you believe research utilisation in your hospital? How do you perceive research utilisation?**

Interviewee response: 20

I believe that there is non-use of research for clinical decision-making practice in our hospital. Nurses and midwives were graduated from universities. They have job description. They do procedures based on job description. For example, I can follow laboring mother using basic emergency obstetric and new born care (BEMONC) check list and partograph. There is also immediate postpartum care and new born care check list to give care for mother and newborn. The quality of health services should be approved through the use of research knowledge.

**2. How do you think the importance of research utilisation? What are important articles used in clinical practice?**

Interviewee response: 20

We know the usefulness of knowledge of research findings. I cannot say nurses and midwives don’t use research in our hospital. We use our effort to search evidences from mobile data and there is discussion about cases among nurses and midwives.

We cannot get guidelines for implementation of evidence based practice. We use our effort to implement evidence based practice. We download books. I can also get books in the form of soft copies from university teachers. But we cannot get any evidences from our hospital.

**3. What are the barriers that you do not utilise research for your clinical decision-making practice?**

Interviewee response: 20

We cannot use single research in healthcare practice. It is difficult to get a systematic review and meta-analysis of research findings. Our hospital has no library and computers. Further, most nurses and midwives didn’t get training to utilise research findings in the healthcare practice

**4. How do you see the supports for research utilisation in your hospital?**

Interviewee response: 20

Case managers, hospital manager, health bureau and non-governmental organizations are helping us by giving training, providing training manuals and guidelines. However, they did nothing in research.

**FGDs**

1**. How do you believe research utilisation in your hospital? How do you understand research utilisation? What is research utilisation mean?**

Participant response: FGD1-01

I am not quite sure to believe the use of research in clinical practice. I use guidelines and other documents as a source of knowledge to help me my clinical decision. I also ask seniors when I am not clear about some procedures. I understand the procedure from my colleagues. I use my colleagues as source of knowledge and skills.

Participant response: FGD1-04

I am not quiet sure about my use of research in clinical decision-making practice. However, the use of research is source of knowledge and it improves ethical related issues. Research utilization is useful for the patients, nurses and midwives. Sharing of knowledge and skills among colleagues are important.

Participant response: FGD1-06

I believe that research utilization is the use of trusted research finding in clinical practice. Use of research in clinical practice shows the direction of activities done during clinical practice. I use guidelines and hospital protocol for this. The use of these guidelines and hospital protocols may not be equivalent to research use.

Participant response: FGD1-10

I have the trust to use knowledge from current research findings. I know that we do not use any source of knowledge blindly. I use updated guidelines. I use training as source of evidences like guidelines and manuals.

**2. How do you think the importance of research utilisation? How do you use research in clinical practice?**

Participant response: FGD1-02

I know it is important. I can an get knowledge from research. For example, evidences or information from patients like malaria distribution can be studied in research so as to use as source of knowledge. Internet is source of evidence. I can get updated information from internet. I know that I can get some procedures directly from internet. I also know that I can get books and guidelines from internet.

I use national guidelines and hospital protocols to improve my knowledge and skills. In addition to this, I read new updated information from internet searching. I use my colleagues as source of evidence.

Participant response: FGD1-03

I know it is good. For example, I can know malaria distribution from research. I also use my colleagues as source of source of knowledge and skills.

Participant response: FGD1-05

Mostly, I use guidelines and hospital protocols in order to order drugs and laboratory request. I use knowledge that I got during school education. I read updated books to update my knowledge and skills. In addition to this; I use research findings for updating my knowledge and skills.

Participant response: FGD1-06

I think research utilization in clinical and healthcare practice improves the success of clinical outcome.

I am not using all research for my clinical decision. I use guidelines and hospital protocols after the responsible bodies provide orientation for these evidences. I cannot use research findings directly from internet because of lack of trustfulness. For example, I use knowledge from research findings after responsible bodies approve it. Ministry of health has different national guidelines. I use it .I also use world health organization guidelines. I believe that as far as evidence is useful for the patient, we can use any source of knowledge and skills.

Participant response: FGD1-07

I know that clinical decision based on knowledge from research has good outcome for the patient. When we see intervention of research based studies of malaria. It has good outcome. Mostly, I use the hospital protocols. I read it every month and I read it even every two days and sometimes.

Participant response: FGD1-08

I know it is important. I can get research article in the form of soft copies. However, I am not quite sure about my knowledge to research findings in clinical practice.

Participant response: FGD1-09

I know it is necessary. However I cannot get trusted research articles. I can get updated guidelines and hospital protocols in the form of soft copies. I get these copies from training or colleagues who get training. I can also get hard copies from training

Participant response: FGD1-12

I know the use of research in clinical practice. I have the perception. For example, when the doctor treat the patient based on the best and current research finding, this is research utilization.

I cannot get current and best research finding to use it in clinical practice. We can get guidelines from ministry of health and world health organization. I use hospital protocols, guidelines and training manuals for my day to day professional practices.

**3. How do you think your / nurses’ and midwives’/ knowledge, skills and attitude towards research utilisation?**

Participant response: FGD1-09

Our handling of research utilization is low. There is gap of skill and awareness. Training of nurses and midwives is important to fulfill these gaps so as to research knowledge in clinical decision making practice

**4. Why don’t you use research in clinical decision practice?**

Participant response: FGD1-03

There are barriers to research utilization. Work overload or work overlap prevent nurses and midwives’ use of research in clinical practice. The other problem is administration issues like loss of freedom in frustration of nurses and midwives by abusing them using attendance follow up to influence them. The other barrier is shortage of logistics like pens and note books.

Participant response: FGD1-07

I do not use research in clinical decision-making practice because of work load and shortage of internet access. The hospital should fulfill internet access, library and other newly updated research findings.

Participant response: FGD1-08

I do not use research articles because of lack of availability.

Participant response: FGD1-09

I do not use knowledge from research because of less interest and motivation. The other reason is the availability of research articles in the hospital. For example, there is not internet access. Lack of perception and attitude is another problem. Research articles should be placed everywhere for nurses and midwives. Work load hinders the use of research in clinical practice. Work load causes tiredness and shortage of time.

There is not internet access in our hospital. I use hard copies of guidelines for implementation of evidence based practice but these are not updated guidelines.

**5. How the supports for research utilisation important?**

Participant response: FGD1-01

I know that there is no encouraging managers’ support regarding to research utilization. There are different challenges of mangers support. For example, salary improvement and incentives are beyond mangers activities

Participant response: FGD1-05

There is no managerial support for research utilization in our hospital. The manager and heads follow which activity is well done and which one is not well done. They do not know what is important for research utilization during clinical decision. There is no way to address the gap of research utilization.

Keeping nurses and midwives’ moral through increasing salary and incentives encourage the use of research findings in clinical decision-making practice. I read books to implement evidence based practice. Reading needs moral and motivation. First of all nurses and midwives should get adequate payment for what they are performing. Reading research is tedious. It needs incentives to motivate nurses and midwives.

Participant response: FGD1-06

Research findings used for clinical decision should be available. For example drug information center should be established. Internet access should be available in all wards. Nurses and midwives commitment is important in spite of the access of evidences. Managers and heads should introduce the use of research in clinical practice through training and discussion during staff meeting. Managers and heads should supervise and control nurses and midwives’ research utilization but they haven’t done it.

I know that managers and heads do not supervise and controls our research utilization. They follow about patient care without evidences. They focus on who provides good care for the patient. They give result based on outcome based services. However, they do not know about research knowledge that can improve clinical decision-making practice.

Participant response: FGD1-11

Managers should supervise and control the use of knowledge from research findings among nurses and midwives. However, our managers and ward heads do balanced score card .They do not encourage research utilization. There should be staff discussion on research utilization in clinical practice. Managers should fulfill resources and access to research utilization.

1**. How do you believe research utilisation in your hospital? How do you understand research utilisation? What is research utilisation mean?**

Participant response: FGD2-01

I believe that research utilization is part of quality health care. There are recent medical articles. I see one study in America that indicate pre-hypertension (130/80 mmHg) in our diagnosis is diagnosed as hypertension in America. We can get such evidences from research articles. Our university is not familiar but I know that there is students’ annual research conference participation in Gondar University. These important research reports were from operation room side, anesthesia, midwives, nurses and others. There were seniors and professors during report presentation. These articles were published and available in the library for health professionals to use it. When I come to our situation, there are not researchers. There is not access of research articles for implementation of evidence based practice. When I get access of evidences, I use it for clinical decision-making practice.

Participant response: FGD2-02

First of all I would like to say thank you for your asking me this information. Best and current research findings solves patients’ problem. I understand that research utilization solves patient problem because we observed better out come on the patient when health professionals use best research findings in clinical decision-making practice.

Participant response: FGD2-03

I understand research utilisation. It is using trusted research findings in healthcare practice. I know nurses and midwives who utilise research findings instead of hospital protocol in the healthcare practice. I use guidelines and hospital protocols to get knowledge and skills for my healthcare decision-making. I utilise research sometimes when I get trusted research findings. I don’t utilise it intentionally

Participant response: FGD2-04

I want to say that the use of best and current research findings free us from accountability when there is patient harm and it is useful for the patient.

Participant response: FGD2-05

Research utilization reduces patient harm, increase professionals knowledge and patient satisfaction when it used in clinical practice properly. It reduces patient harm, increase professionals knowledge and patient satisfaction

Participant response: FGD2-06

I think research utilization improves quality care service and patient satisfaction

Participant response: FGD2-07

I believe that research utilization improves patient harm and accountability. It also increases patient satisfaction and it helps for the achievement of the objectives of our hospital

Participant response: FGD2-08

Research utilization determines quality care for the patient and patient satisfaction.

Participant response: FGD2-10

I know the time is revolution for research utilization .I understand that we have to use knowledge from research in clinical practice.

**2. How do you think the importance of research utilisation? How do you use research in clinical practice?**

Participant response: FGD2-03

There are different guidelines, research articles, and hospital protocols that are used in clinical decision. One should provide health services based on these points in clinical practice. I see that most staffs do not use updated guidelines and research articles in spite of using text books and school knowledge. This is also probability. I use my previous knowledge and skill in clinical practice.

Participant response: FGD2-04

Scientific evidences like best research findings, guidelines and hospital protocols are used in clinical practice.

Participant response: FGD2-05

There are training manuals and guidelines in our ward that we use in clinical decision-making practice. In addition to this, trained staffs share ideas about the new training manuals so that we can use the new manuals in clinical practice. The doctors also share their idea and we use it for source of knowledge and skills.

Participant response: FGD2-06

I use guidelines and training manuals like basic emergency obstetric and new born care (BEONC) in clinical decision-making practice. Research study result also important for clinical decision but I do not use it.

Participant response: FGD2-07

I have perception and knowledge about guidelines’ use in clinical and healthcare practice. We do not read research articles for the sake of clinical decision. Our hospital staffs use guidelines in clinical and healthcare. However, the senior doctors tell us about research results to it in clinical and health care practice.

Participant response: FGD2-08

I think we have the knowledge and perception of research articles but we do not update ourselves for new knowledge.

W e can get evidence from different ways. There is recommendation to use research evidence. For example, if caesarean section is done for high number HIV viral load cases, it decrease mother to child HIV transmission. This is the evidence that we can get from research. I use these knowledge from research. This information is useful for the patient.

Participant response: FGD2-09

Research utilization is professional obligation. I get best and current research findings from internet. We can also get research articles when we are interested but we do not use it for clinical decision. I use guidelines prepared by seniors.

Participant response: FGD2-10

Most of the time, we use national guidelines and hospital protocols for our clinical decision. In addition to this school education and research articles are useful for clinical practice.

There are research articles from internet. I use this for my knowledge but not for clinical decision. Even though, there is not training, we can download research articles, health policies and national guidelines and use these evidences for clinical decision.

**3. How do you think your / nurses’ and midwives’/ knowledge, skills and attitude towards research utilisation?**

Participant response: FGD2-03

There are factors that hinder the use of research knowledge in clinical decision-making practice. There is fear of research finding when they use it because of harmfulness for the patient. The other factor is lack of awareness and knowledge. We do not believe research article in clinical practice.

**4. Why don’t you use research in clinical decision practice?**

Participant response: FGD2-02

There are barriers to use research in clinical practice. These are lack of access to research articles, internet, and other resources. I do not use internet because it harms my eye. The other barrier is the hospital library is far from ward. Moreover, it has no space and tables for reading materials in the library. Many people cannot use the library at the same time.

Experienced colleagues are limited to their ward. There is rotation of staff every year. When experienced staffs work in one ward for long period of time, he/she become master for that particular activity. Because of rotation the staff loss his experience and they are like fresh for their new ward. The other problem is lack of attitude and motivation of staff to share their experience.

Participant response: FGD2-03

Although we have the knowledge of research findings, we do not give attentio.The other problem is increasing patient flow which causes lack of time to read research finding.

Participant response: FGD2-04

There are factors that influence health professional to use knowledge from research finding in clinical decision-making practice. These are imbalance of health service provider and patient flow ( work load), lack of internet access and limited access of research used in clinical decision to each ward.

Participant response: FGD2-05

There are barriers for research utilization in our hospital. The guidelines and training manuals are present in different ward. I am in neonatal intensive care unit ward and I do not know how to use my knowledge of research findings in clinical practice. Even I do not know the reason. The problem may be library or communication. In spite of this problem, I use guidelines and manuals of my ward to decide my practical activities..

Our hospital has not desk tops for each ward as other hospitals have this. The desk tops are in our managers or head office and we cannot get these computers. This prevents the staffs to get best research finding from internet in clinical practice.

Participant response: FGD2-08

When I see practical procedures, we do not follow the steps of procedures or protocol because of high patient flow. Our work is to give health care for all patients who come to our hospital.

Participant response: FGD2-10

There are factors that influence research utilization among nurses and midwives. These are lack of internet access and shortage of time. In my hospital, there is limited access of internet. I believe that nurses and midwives do not use internet during their free time. There is also limitation to use library. There are few books in the room of library. These are not enough for nurses and midwives.

**5. How the supports for research utilisation important?**

Participant response: FGD2-02

Relating to managerial support, there is monitoring and supervision. There is quality care team which observes daily, weekly and monthly activities of staffs. The team encourages best performance of the staffs and provides support for poor performance of the staffs. Beyond this, managers say this activity is done, this is not done, you are not punctual and you are absent but they do nothing about research articles to use its knowledge for clinical decision-making practice.

Participant response: FGD2-03

My hospital has a protocol to control diabetic ketoacidosis. We use this protocol. I use one research finding that showed the treatment dose of insulin was based on the weight of the patient. Diabetic Mellitus was controlled immediately when I compared it with our hospital protocol. I did it by myself. It is not official. But our protocol does not use the treatment based on patient weight and it takes time to control ketoacidosis. We have fear in using research because of harmfulness to the patient. There should be trust to use it. Do you see here? I didn’t get any training. We do not have a computer. What do you mean? There should be a supporter to give us confidence in using research. We are dependent on hospital protocols and guidelines.

Participant response: FGD2-06

Non-governmental organizations supervise, control and support us to use training manuals, guidelines and protocols in our clinical decision. However, our manager and ward heads do not follow us how to use it in clinical practice. Research utilization is absent totally.

Participant response: FGD2-07

I see there is managerial supportive supervision in our ward. For example, there is quality improvement team which follows day to day activity of health service providers. The team has its own project to give encouraging and supportive feedback.

Participant response: FGD2-10

Mangers and head of wards do not follow nurses and midwives weather they use research articles or not. They do not have supportive supervision of implementation of evidence based practice. I have not seen any mangers or heads who encourages the use of research articles and books.

1**. How do you believe research utilisation in your hospital? How do you understand research utilisation? What is research utilisation mean?**

Participant response: FGD3-01

I believe that research utilization has good impact of quality healthcare .It is professional person’s knowledge and skill development through reading best research findings and apply the information for his professional practice

Participant response: FGD3-02

Research utilization means clinical practice based on research and follows scientific procedures to provide health care services for the clients.

**2. How do you think the importance of research utilisation? How do you use research in clinical practice?**

Participant response: FGD3-01

I have not use books and read any research articles for my clinical practice. I use my colleagues and national guidelines as a source of knowledge for my professional practice.

Participant response: FGD3-02

There are many evidences used as sources of knowledge, skill and attitude. At first level one is expected to get his knowledge from college or university .At this level, the evidences are his teacher, books, and internet. Guidelines are evidences obtained from internet

The use of evidence based practical decision is to minimize mistakes, to update our information, to increase our knowledge, to get good result and to improve accountability. In addition to this, evidence based practice improves quality health care services.

For all discipline, there are documented hard copies of guidelines. These evidences are distributed to all wards so the professionals refer these evidences for clinical practical decisions. Moreover, referral books , research articles and other evidences to each discipline are available in all wards. When the professionals are not clear about their decision, they use these evidences. I also use these evidences sometimes, when I am not clear about my decision.

Most of the time, I use nursing standards for evidence based practice. I use this standard for nursing diagnosis. Even if, there is on and off library in our hospital, we refer some books. It is possible to get book by borrowing it from hospital library. Regarding to research article, I did not use it. However, we know our job is team work, there is supporting and sharing of idea between team members. I use health professional for my evidence based practice.

Participant response: FGD3-03

We have updated national guidlinesand world health organization guidelines. These are important for quality health care. I use mass media and my colleagues to get knowledge. However, I have no idea on research utilization.

Participant response: FGD3-04

I use books, guidelines and my colleagues as a source of knowledge for my clinical decision. We can get published evidences like books and guidelines from health bureau and our hospital in the form of hard copies. We use soft copies by sharing through our mobile. But there is not access of soft copies. I can get research article. Our colleagues share their knowledge to me during morning session recently. Previously we used 1 to 5 group discussion instead of morning session.

Participant response: FGD3-05

I know implementation of evidence based practice means the use of written documents obtained from universities, internet and trainings.

I know that selection of best evidences to use in clinical and healthcare practice is important to give quality care for the patients. I can knowledge and skills from my colleague and organizations that give training for us. I can get guidelines, books and research articles from internet.

Participant response: FGD3-06

All things were mentioned. I have points that are not discussed. In our job, there is always updated knowledge and skill. Through training, we can get updated knowledge and skill. Guidelines and training manuals are important for implementation of evidence based practice. In addition to this, we can use research articles and different books.

Participant response: FGD3-07

W e could not do our work without knowledge and skills. Rese4arch finding is one of the sources of knowledge and skills. If we do our procedures based on knowledge from research, we are free from accountability due patient harm. Research utilization is useful for patient safety and quality health care.

Participant response: FGD3-08

I know that research utilization is the use of knowledge from research findings in clinical decision during procedures. I use training manuals and guidelines .I have not time to use books and research articles in the hospital. I use soft copy to read at home. In addition to this I use my colleagues as source of knowledge.

**3. Why don’t you use research in clinical decision practice?**

Participant response: FGD3-01

Nurses and midwives have not got learning opportunity. We tried to improve our skill using evidence .Moreover, research utilization increase if there is plan for change of level of our education. Plan for change of level of education encourages nurses and midwives to research knowledge in clinical practice. Because of lack of learning opportunity, we are influenced to update ourselves using knowledge from research findings.

Participant response: FGD3-02

The managers do not respect hospital workers equally. It becomes a culture. There is a problem with the hierarchy. From the top, there are senior doctors. At the bottom level, there are janitors and guards. The attitude of managers to respect their workers decreases from top to bottom. Even if, nurses and midwives get evidence from research and try to utilise it, nobody accepts us. How do you feel this kind of demoralization? Ohm… I cannot explain it.

Participant response: FGD3-06

I do not utilise research for my clinical practice. I always do the same procedure and my colleagues too… I feel frustrated. Nothing is encouraging. Sorry to say this. I thought that there is no need to use research for these repeated activities in the clinical setting.

Participant response: FGD3-07

Nurses and midwives do not use research in clinical practice because of lack of source of evidences like internet access .The other is attitude problem of nurses and midwives. We have the idea of we know all things. No one knows more than me. Even when we do not know about one thing, we fear to ask others because of criticize our knowledge. In this case we do procedures without knowledge and skills.

Participant response: FGD3-09

There is a very great workload. I cannot read anything during working hours. I cannot read even at home. After working hours, I go home and I want to sleep due to tiredness. It is impossible to go to the library and read an article and it is impossible to search journals due to lack of time. I have to get time either during working hours or at home to search and read journals related to our health service.

**4. How the supports for research utilisation important?**

Participant response: FGD3-04

There is no hospital manager support to use knowledge from research findings in clinical and healthcare. They are interested in taking attendance. They simple say who is late and absent. The managers do not have care about quality health care services. Their concern is how many of the patients get health service. Their concern is quantity rather than quality.

Participant response: FGD3-07

Managers’ support is very low to use research in clinical and healthcare practice. It is better to discuss on the research findings before using in clinical practice.

Participant response: FGD3-09

Relating to research utilization, our hospital has problem. It is not common to motivate nurses and midwives.

There is no supportive supervision towards the use of research in clinical practice. In this hospital, there is very great work load and I cannot read anything during working hour and at home. After working hour I go home and I sleep because of tiredness. There is no internet access in this hospital.

1**. How do you believe research utilisation in your hospital? How do you understand research utilisation? What is research utilisation mean?**

Participant response: FGD4-01

I believe that research utilization is advantageous for the patient and health service providers. It has great value for quality health service. We perform our activities based on knowledge and skill obtained from different evidence in this hospital.

Participant response: FGD4-06

I understand that research utilization is useful for development health service provider, improvement of quality health service for the patients and service provision based on ethics. There is failure of research utilization. There are different problems.

Participant response: FGD4-08

**2. How do you think the importance of research utilisation? How do you use research in clinical practice?**

Participant response: FGD4-02

Knowledge and skills from research is always updated. Some of nurses and midwives should get these research findings timely. Individual activities decide to get research articles. Some of nurses and midwives use outdated evidences. Research utilization is useful for the patient, hospital and health service providers.

Participant response: FGD4-03

We do not use research articles in clinical and healthcare in clinical practice. It is not feasible to use knowledge from research findings in clinical practice

Participant response: FGD4-04

Unless ministry of health distributed research result in the form of guidelines, we do not use it. We can use research findings when we are sure of the harmlessness for our patients. However, I cannot use these knowledge from research findings in clinical practice individually. The hospital can use these evidences after creating awarnes for all health service providers.

**3. How do you think your / nurses’ and midwives’/ knowledge, skills and attitude towards research utilisation?**

Participant response: FGD4-04

I do have concern on my knowledge and skill to use research in clinical practice. Most of us cannot interpret the result part of articles. There is problem to understand word in the articles.

**4. Why don’t you use research in clinical decision practice?**

Participant response: FGD4-01

Nurses and midwives do not use research in clinical practice because of lack of attention, lack of interest to use trusted and current articles, problem of skill, lack of satisfaction, lack of motivation, lack of access of internet, lack reading room, work overload, lack of computer, lack of library, lack of skill lab demonstration, resistance to changes and lack of accountability

Participant response: FGD4-05

Nurses and midwives do not use research in clinical and healthcare practice because of lack of library and reading room, work overload, lack of internet access, lack of computer in nurses and midwifery stations, lack of interest, lack of access of best and current research findings and fear of harmfulness of knowledge and skill from research.

Participant response: FGD4-06

Hospitals should find research articles from different sites. There is not this kind of system. I am not quite sure of this communication. I think there is break between hospital and health bureau and ministry of health. There is need of creating awareness of nurses and midwives.

Participant response: FGD4-07

The working time of the library should be known for nurses and midwives. When one leaves this hospital, he cannot get the library workers for signature of clearance.

**5. How the supports for research utilisation important?**

Participant response: FGD4-05

There are stake holder like quality unit officers, managers, non-governmental organizations and others. Quality unit health officers support the training of nursing care plan. Non governmental organizations support our hospital on training of family planning. However, there no stakeholders that support research utilization in clinical and healthcare practice

Participant response: FGD4-08

There is not supportive supervision and controls of implementation of evidence based practice of nurses and midwives

**Observation**

**1. Did the hospital have internet and library?**

H01

**No**

**How do you describe this?**

There is not any internet and library access in the hospital.

H02

**No**

**How do you describe this?**

The hospital has not any internet and library access.

H03

**No**

**How do you describe this?**

There were not any internet access and library in the hospital.

H04

**No**

**How do you describe this?**

There were not any internet access and library in the hospital.

H05

**Yes**

**How do you describe this?**

There was internet access in the hospital.

H06

**Yes**

**How do you describe this?**

There was internet access in the hospital.

H07

**No**

**How do you describe this?**

There were not any internet access and library in the hospital.

H08

**Yes**

**How do you describe this?**

There were internet access, library in the hospital and it is not comfortable

**2. Did nurses and midwives have adequate and comfortable working room?**

H01

**Yes**

**How do you describe this?**

Nurses and midwives have adequate and comfortable working room and there are guidelines in most of wards.

H02

**Yes**

**How do you describe this?**

Nurses and midwives had not comfortable and adequate working room. More than eight nurses and midwives worked in small room which cannot accommodate more than three nurses or midwives in some working units.

H03

**No**

**How do you describe this?**

There was no comfortable and adequate classroom for the staff in labor ward, injection and dressing room, antenatal care unit and outpatient ward.

H04

**No**

**How do you describe this?**

There was no comfortable and adequate classroom for the staff in labor ward, injection and dressing room, antenatal care unit, under five child health care unit and outpatient ward.

H05

**No**

**How do you describe this?**

There were no library and comfortable and adequate class room for the staff in labor ward, injection and dressing room, antenatal care unit, under five child health care unit and outpatient ward.

H06

**No**

**How do you describe this?**

There were no library and comfortable and adequate class room for the staff in labor ward, injection and dressing room, antenatal care unit, under five child health care unit and outpatient ward.

H07

**Yes**

**How do you describe this?**

There was comfortable and adequate class room for the staff in labor ward, injection and dressing room, antenatal care unit, under five child health care unit and outpatient ward.

H08

**Yes**

**How do you describe this?**

There were comfortable and adequate class room for the staff in all wards like labor ward, injection and dressing room, antenatal care unit, under five child health care unit and outpatient ward.

**3. Did nurses and midwives use research articles in clinical decision?**

H01

**No**

**How do you describe this?**

They use guidelines during counseling and advice of the clients and practical procedures. There are infection prevention guidelines and neat infection prevention techniques in the labour ward, injection and dressing room, minor and major operation room. However, nurses and midwives has no idea to use research articles in clinical practice

H02

**No**

**How do you describe this?**

Nurses and midwives has no idea to use research articles in clinical practice. However, they had guidelines and they use guidelines for counseling and advice during procedural activities.

H03

**No**

**How do you describe this?**

There were guidelines in each ward and nurses and midwives use the guidelines for counseling and advice of client. There was infection prevention guideline in injection room but they did not use the guideline to implement infection prevention techniques. However, nurses and midwives has no idea to use research articles in clinical practice

H04

**No**

**How do you describe this?**

There was no comfortable and adequate classroom for the staff in labor ward, injection and dressing room, antenatal care unit, under five child health care unit and outpatient ward.

H05

**No**

**How do you describe this?**

Nurses and midwives had guidelines and they did not use knowledge from research articles, guidelines during procedural activities. Infection prevention techniques were neat and clear in all wards and units. They did not use guidelines for counseling and advice.

H06

**No**

**How do you describe this?**

Nurses and midwives had not guidelines and they did not use knowledge from research articles and guidelines during procedural activities and counseling and advice of client. Infection prevention techniques were not neat and clear in all wards and units. Midwives use partograph during laboring mother follow up appropriately to help them for decision.

H07

**No**

**How do you describe this?**

Nurses and midwives had guidelines. Infection prevention techniques were neat and clear in all wards and units. However, they did not use guidelines during procedural activities and counseling and advice of client. Nurses and midwives also has no idea to use research articles in clinical practice.

H08

**No**

**How do you describe this?**

Nurses and midwives had guidelines and they use guidelines during procedural activities and counseling and advice. But infection prevention techniques in delivery ward was not neat and clear.

**4. Do nurses and midwives fill working documents like partograph based on the standards?**

H01

**Yes**

**How do you describe this?**

Nurses and midwives documented procedures based on the standards and checklists obtained from evidences like partograph which is important for follow up of laboring mothers for decision.

H02

**Yes**

**How do you describe this?**

Midwives use partograph during laboring mother follow up appropriately to help them for decision. But most of nurses and midwives did not use guidelines for their practical activities. There is not any infection prevent guidelines in minor operation room.

H03

**No**

**How do you describe this?**

Nurses and midwives did not use the available guidelines for implementation of evidence based practice in all wards and units. Midwives did not document partograph appropriately. They argued that there was no need to record second stage of labor events on the partograph.

H04

**Yes**

**How do you describe this?**

Midwives use partograph during laboring mother follow up appropriately to help them for decision. There were not any infection prevent and injection guidelines in minor operation room and injection and dressing room.

H05

**Yes**

**How do you describe this?**

Midwives use partograph during laboring mother follow up appropriately to help them for decision.

H06

H07

**Yes**

**How do you describe this?**

Midwives use partograph during laboring mother follow up appropriately to help them for decision.

H08

**Yes**

**How do you describe this?**

Midwives use partograph during laboring mother follow up appropriately to help them for decision. There were not any infection prevent and injection guidelines in minor operation room and injection and dressing room.

**5. Do nurses and midwives perform their competencies without difficulty?**

H01

**No**

**How do you describe this?**

The observation of practical procedures using check lists like basic emergency obstetric. The observation indicated that nurses and midwives perform the procedures with difficulty.

**No**

**How do you describe this?**

The observation of practical procedures using check lists like basic emergency obstetric. The observation indicated that nurses and midwives perform the procedures with difficulty.

H03

**No**

**How do you describe this?**

Three procedures were observed using checklists like basic emergency obstetric and newborn care, family planning and injection guidelines checklist. The observation indicated that nurses and midwives perform the procedures with difficulty.

H04

**No**

**How do you describe this?**

Three procedures were observed using checklists like basic emergency obstetric and newborn care, family planning and infection prevention checklist. Most of the procedures were performed without referring best and currently available research findings. The observation indicated that nurses and midwives perform the procedures with difficulty.

H05

**No**

**How do you describe this?**

Three procedures were observed using checklists like basic emergency obstetric and newborn care, family planning and infection prevention checklist. Most of the procedures were performed without referring best and currently available research findings. The observation indicated that nurses and midwives perform the procedures with difficulty.

H06

**No**

**How do you describe this?**

Three procedures were observed using checklists like basic emergency obstetric and newborn care, family planning and infection prevention checklist. Most of the procedures were performed without referring best and currently available research findings. The observation indicated that nurses and midwives perform the procedures with difficulty.

H07

**No**

**How do you describe this?**

Three procedures were observed using checklists like basic emergency obstetric and newborn care, family planning and infection prevention checklist. Most of the procedures were performed without referring best and currently available research findings. The observation indicated that nurses and midwives perform the procedures with difficulty.

H08

**No**

**How do you describe this?**

Procedures like labour and delivery, implanol insertion, injection and dressing were observed using checklists like basic emergency obstetric and newborn care, family planning, infection prevention and injection and dressing checklist. Most of the procedures were performed without referring best and currently available research findings.. Most of the procedures were performed without referring best and currently available research findings. The observation indicated that nurses and midwives perform the procedures with difficulty.

**All engaging and probing questions including checklist used for data collection**

**FGD related Questions for the study**

**Engaging questions**

1. How do you believe research utilisation in your hospital? How do you understand research utilisation? What is research utilisation mean?

**Probing questions for question number 1**

What do you mean believing of research utilisation? Do you have any information before? How do you think nurses/ midwives believe towards research utilisation?

1. How do you think the importance of research utilisation? How do you use research in clinical practice?

**Probing questions for question number 2**

What is your experience of research utilisation in clinical practice? What are the articles used for your clinical decision making practice? How research could be used in clinical decision making practice? How do you get trusted articles?

1. How do you think your / nurses’ and midwives’/ knowledge, skills and attitude towards research utilisation?

**Probing questions for question number 3**

How do you explain the knowledge and skill of nurses and midwives experience of research utilisation? Do you have the knowledge and skill to utilise research in clinical decision-making practice? How do you elaborate your knowledge and skill to utilise research? What is your experience regarding to research utilisation in your hospital? How do you share your experience of research utilisation?

1. Why don’t you use research in clinical decision practice?

**Probing questions for question number 4**

What are barriers of research utilisation? Which barriers are common in your hospital? How these barriers affect your clinical decision making practice? How do you overcome these barriers?

1. How the supports for research utilisation important?

**Probing questions for question number 5**

What are the supports for research utilisation in your hospital? Who are the supporters for research utilisation? How do you see the support of your managers’ role to use research in clinical practice? Why do not managers support research utilisation? How do you describe the supportive supervision, monitoring and evaluation of research utilisation in your hospital?

**Interview related questions for the study**

**Engaging questions**

1. How do you believe research utilisation in your hospital? How do you perceive research utilisation?

**Probing questions for question number 1**

What is your believe towards research utilisation? How do you explain your experience of research utilisation in your hospital? How do you use research? What are articles used in clinical practice? What are these articles? How these research articles could be used? How do you get trusted research? How do you explain your knowledge and skill to utilise research? Do you have the knowledge and skill to utilise research? How do you elaborate your knowledge and skill to utilise research?

1. How do you think the importance of research utilisation? What are important articles used in clinical practice?

**Probing questions for question number 2**

Do you utilise research for clinical decision-making practice? How do you get trusted articles? Do you think that research utilisation improves quality health care? Do you think the importance of research utilisation? How it is important?

1. What are the reasons that you do not utilise research for your clinical decision-making practice?

**Probing questions for question number 3**

What are barriers of research utilisation? Which barriers are common in your hospital? How these barriers affect your clinical decision making practice? How do you overcome these barriers?

1. **How the supports for research utilisation important?**

**Probing questions for question number 4**

What are the supports for research utilisation? Who are the supporters of research utilisation? How do you see the support of your managers’ role for research utilisation? Why do not managers support research utilisation? What is your contribution to support research utilisation? How do you describe the supportive supervision, monitoring and evaluation of research utilisation in your hospital? How do you explain the supportive supervision, monitoring and evaluation of research utilisation in your hospital?

**Participatory observation related check-list for the study**

Place a “√” in box if research is utilised and facilities are available satisfactorily, an “**X**” if it is **not** utilised **satisfactorily**, or if not observed.

| Observation check list | Mark |
| --- | --- |
| 1. Midwives /nurses utilise research during procedures in clinical practice |  |
| 1. Apply infection prevention based on research findings |  |
| 1. Provide counseling about necessary topics based on research findings. |  |
| 1. The hospital has library. |  |
| 1. The hospital has research articles |  |
| 1. The hospital has internet access |  |
| 1. The hospital has adequate work room for nurses/midwives |  |
| 1. There is access to a system for comprehensive literature searching to utilise systematic review and meta analysis. |  |
| 1. Midwives/nurses perform their competencies without difficulty? |  |

- **Describe all the observed events during data collection based on the check-list**
